# Using 17^th^ century medication for modern diabetes management: Doctors’ perceptions of self-medication practices – A qualitative study

**DOI:** 10.1007/s40200-022-01154-5

**Published:** 2022-11-12

**Authors:** Rahul Krishna Puvvada, Clarice Y. Tang, Jency Thomas, Mitch Kay, Peter Higgs, Markandeya Jois, Ramesh Madhan, Sabrina Gupta

**Affiliations:** 1 Department of Microbiology Anatomy Physiology and Pharmacology (MAPP), School of Agriculture Biomedicine and Environment (SABE), La Trobe University, Melbourne, Australia; 2 Department of Pharmacy Practice, JSS College of Pharmacy, JSS Academy of Higher Education and Research, Mysuru, Karnataka India; 3 Department of Physiotherapy, School of Health Sciences, Western Sydney University, Sydney, Australia; 4 Department of Public Health, School of Psychology and Public Health, La Trobe University, Melbourne, Australia; 5 Allied Health Department, South Western Sydney Local Health District, Sydney, Australia; 6 Burnet Institute, Melbourne, Australia

**Keywords:** Doctors, Perception, Type 2 Diabetes, Self-Medication, India

## Abstract

**Purpose:**

This study was conducted to explore doctors’ perceptions and understanding of the self-medication practices of people living with type 2 diabetes.

**Methods:**

A qualitative research design incorporating 20 semi-structured, face-to-face interviews were conducted with doctors treating people with type 2 diabetes in Mysuru, India, between July 2019 and January 2020. All the interviews were conducted in doctors’ clinics, audio-recorded and thematically analyzed.

**Results:**

Three themes were identified from these interviews- i) Doctors’ beliefs towards their patients’ use of traditional medicine and environmental factors influencing prescription practices, ii) Doctors reported little faith in traditional medicines, iii) Limited strategies implemented by doctors to overcome barriers to self-medications. Doctors reported greater belief in western medications over traditional medications and expressed concern that their patients favored traditional medications over western. Multiple factors such as social media, accessibility of healthcare facilities and pill burden influenced adherence to western medications. Also, lack of knowledge about traditional medications and trust in western medications available under government schemes have influenced prescription practices among doctors. It appears that doctors implemented strategies such as educating patients on the detrimental effects of self-medication and insisting on patients to take only western medications to achieve desired blood glucose levels when managing self-medication practices among people with diabetes.

**Conclusion:**

These results suggest that doctors have limited strategies to implement to prevent self-medication practices among people with diabetes. Increasing knowledge amongst doctors about JAS medication effectiveness and thereby garnering greater trust in generic medications. In addition, efforts should be made to identify the best ways to integrate traditional and western medicine into patient-centered care delivery.

**Supplementary Information:**

The online version contains supplementary material available at 10.1007/s40200-022-01154-5.

## Introduction

Type 2 diabetes is the most common type of metabolic disorder, accounting for 90% of the world's diabetes population [1]. According to International Diabetes Federation, in 2021, nearly 81% of people living with diabetes were from low and middle income countries and that one in seven people living with type 2 diabetes worldwide were from India [1]. This diabetes burden on Indian population might be due to multiple factors such as genetics, sedentary lifestyle changes and urbanization [2]. Furthermore, poorer levels in health literacy among people with diabetes in India also impact on the effectiveness of self-management practices, which in turn impact on the management of diabetes [2].

Optimal management of diabetes includes continuous pharmacological treatment and lifestyle modifications such as regular physical activities, weight reduction and following diabetes-specific diet. However, studies show that adherence to both lifestyle recommendations [3] and pharmacological treatment is difficult and remains sub-optimal due to increases in the pill burden, inadequate financial resources, chronicity of the disease and prolonged treatment regimen [4]. Findings from a recent systematic review found that over half (56%) of people living with type 2 diabetes in India self-medicate by discontinuing western medications and/or consuming traditional medications, and they perceive that their diabetes can be cured through traditional medications [5].

Self-medication is the act of taking medications, herbs or home remedies on one's own initiative, or on another person's advice, without medical advice [6]. Adopting self-medication practices in chronic disease management is a concern due to increase in risk of overuse of medications, misdiagnosis and adverse effects as well as increased the financial burden due to unscheduled hospital visits [7].

While self-medication practices can potentially result in detrimental effects on people with diabetes, previous studies conducted in India and other contexts globally amongst people living with type 2 diabetes found that fear of experiencing adverse effects and high cost from western medications are factors affecting their self-medication practices [5, 8–11]. But in addition, many people with type 2 diabetes in India continue to perceive traditional medications as safe and effective in the management of diabetes [5, 8, 12, 13]. Similar findings were observed in a South-Asian study where the perceived benefit of traditional medications and side effects to western medications contributed to self-medication practices and non-adherence to anti-diabetes medications among people living with type 2 diabetes [14].

Previous studies found that such self-medication practices are also seen among India diaspora migrated to western countries where people living with type 2 diabetes procure traditional medications from their friends and family members in India, suggesting that self-medication practices among people from India remain widely practiced regardless of migration [15, 16]. This perception of traditional medications and use of traditional medications as self-medication are widely practiced in India, and such practices can be dangerous due to the increased risk of misdiagnosis, irrational use of medications and the work burden this can create for doctors [5, 8, 12, 13]. As doctors are the primary care providers for people living with type 2 diabetes, it is necessary to understand doctors’ perceptions towards patients’ medication taking behaviour and self-medication with traditional and/or western medications.

While there is current literature exploring perceptions of self-medication practices among people with diabetes, there are no current studies we are aware of that are exploring doctors’ perceptions of self-medication practices focusing on people with diabetes from India. Therefore, this study aimed to explore the doctor’s perceptions and experience in managing self-medication practices among people living with type 2 diabetes. Improving our understanding on this is important to assist in developing patient-centered strategies that address issues related to self-medication practices*.*

## Methods

To explore the perceptions of doctors about factors that influence self-medication practices among people living with type 2 diabetes, we adopted a phenomenological qualitative research design utilizing semi-structured interviews [17]. The COREQ guidelines were used to represent the findings of the study [18]. Human research ethics approvals were obtained from the Human Ethics Committees of La Trobe University (HEC19227) and JSS Academy of Higher Education and Research (JSSMC/IEC/1107/10NCT/2019–20).

Data collection for this study was conducted between July 2019 and January 2020 in urban Mysuru (Karnataka state in the south-west of India) [19]. The healthcare system of India is a mix of both public and private healthcare providers primarily focusing in rural and urban areas respectively [20]. Irrespective of their financial status, people in India prefer to visit a private clinic or tertiary care hospitals to manage their type 2 diabetes [20, 21]. These clinics and hospitals are being managed by general practitioners or endocrinologists or diabetologists [21]. Therefore, doctors specializing in internal medicine and working as general practitioners or endocrinologists or diabetologists in tertiary care hospitals or private clinics in Mysuru were included in this study via flyers, emails and snowball sampling.

Interested participants contacted members of the research team (RKP or SG), who provided detailed information about the study. RKP then followed up with these participants to obtain informed written consent before determining a mutually suitable date, time and location for the interview. Being local to Mysuru, RKP was aware of local culture and this insider status enhanced trust and rapport building between the interviewer and the participant. Consent was provided by participants before starting the interview. During the interview, participants were also asked to complete a demographic form (Appendix 1). All interviews which were conducted in English, audio-recorded in the doctors’ clinics. No language assistance was required for the interview because all the participants were fluent in English.

The research team RKP, SG and JT prepared the semi-structured interview guide (Appendix 2) based on existing literature [5] and subject experts. This guide included questions exploring the doctor’s perceptions of self-medication practices among patients with type 2 diabetes, the doctor-patient relationship and how doctors manage self-medication practices. A pilot interview was conducted with two researchers in the field of diabetes self-management to further refine the questions prior to conducting the first interview. No major changes in the interview guide were made. Therefore, data from these pilot interviews were included in the analysis. To enhance the trustworthiness of the data, Lincoln and Guba trustworthiness criteria was used [22]. All interviews were transcribed verbatim before being sent to the participants for member checking (also known as respondent validation) to increase the credibility and confirmability of the study [23]. The transferability was ensured by providing detailed description of the context, location of interviews conducted and type of participants involved. Dependability was affirmed by conducting peer-debriefings on the study methods such as process of recruitment of participants. Reflexivity influenced by the research team’s prior experience in conducting diabetes related studies. Principles of theoretical saturation were used to guide data collection and when no new insights were offered. RKP analysed the interviews for point of saturation which was achieved when no new codes were generated [24].

The transcripts were first reviewed in their entirety for codes by the individual members of the research team, RKP (a pharmacist by profession with experience in communicating with doctors and patients) and MK (an experienced qualitative researcher). The inclusion of an insider (RKP) and outsider (MK) to this study enhanced the trustworthiness of the data analysis process. To ensure inter-rater reliability [25], these two researchers independently performed the coding and thematic analysis of the transcripts with the assistance of NVIVO-12 [26]. All the data were analysed using Braun and Clarke's step-by-step guide for thematic analysis [27]. After completing the initial coding, RKP and MK compared their analysis for consensus and subsequently met with SG (an experienced qualitative researcher) to discuss the codes and identify possible themes. A discussion with the entire team then determined the final themes and sub-themes for the study.

## Results

Of 28 doctors who expressed interest in participating in the study, 20 doctors were interviewed. The eight doctors declined to participate because of a lack of availability. The mean duration of interviews was 31.4 ± 10.9 min (range 15 to 53 min). Half the doctors (n = 10) were working in hospital settings, with the others working in a community setting (Table 1).

**Table 1:** Participant demographics (*n* = 20)

Characteristics of participants	Frequency	Percentage
Age (years)		
Mean age ± SD	47 ± 12.3	-
30–39	7 (35)	35
40–49	4 (20)	20
50–59	5 (25)	25
≥ 60	4 (20)	20
Gender		
Male	18 (90)	90
Female	2 (10)	10
Workplace setting		
Hospital	10 (50)	50
Community	10 (50)	50
Years of experience		
0–9	4 (20)	20
10–19	5 (25)	25
20–29	3 (15)	15
30–39	7 (35)	35
≥ 40	1 (5)	5

All doctors were general physicians with a patient caseload that comprised largely of people with type 2 diabetes. Three key themes were identified after the thematic analysis of the interviews which describes the perceptions of doctors towards people living with type 2 diabetes and their self-medication practices, the barriers faced by doctors in the management of type 2 diabetes and the strategies implemented to overcome the barriers. These themes are i) Doctors’ beliefs towards their patients’ use of traditional medicine and environmental factors influencing prescription practices, ii) Doctors reported little faith in traditional medicines and iii) Limited strategies implemented by doctors to overcome barriers to self-medications. The data used to generate themes and subthemes are presented in the supplementary material (Appendix 3).

### Doctors’ beliefs towards their patients’ use of traditional medicine and environmental factors influencing prescription practices

In general, doctors described the two most common types of self-medication practices adopted among patients. The first was to continue using prescribed western medications beyond the validity of the prescription.

*“…As I said, maximum prescription I will give is for three months. I will tell patient that even if diabetes is stable or have no complaints, still you need to do your sugars at least once in three months and meet. But, lot of patients will not come, they will come once in four months, six months, sometimes even after one year, and many patients will continue taking those [previously prescribed] medications.”( P-07, Male)*

The second was the discontinuation of the prescribed western medications and consumption of traditional medications without formal advice from a registered medical practitioner.

*“One patient was on allopathic [western] medications have very good control of HbA1c around 7.5%...saw some TV ads…got tablets [traditional medications] from online and completely stopped the allopathic [western] medication for 3 months… came to me after the HbA1c was nearly 13%.” (P-11, Male)*

Doctors perceived that the increased faith towards self-medication practices and use of traditional medications can be due to multiple different factors.

- **Patients’ poor knowledge about the disease and medications**

Some doctors (*n*=7) said that their patients had limited knowledge of diabetes management and the related complications that may eventuate from uncontrolled diabetes. Doctors mentioned that many patients perceived that diabetes could be managed without medications.

*“There are patients who will be only on diet, exercise…sugars will be under control without medications…It is possible. But only in the initial phase [of diabetes]. Somebody with long-standing diabetes, it will not work.” (P-07, Male)*

Apart from poor knowledge of the disease, doctors believed that patients they were treating also had limited knowledge about western medications. Half of the participants (*n* = 10) also reported that fear of pain from injections and together with the patient’s belief that insulin should only be used as a last option, largely contributed to their avoidance of insulin.

*“…I tell them in the first visit only that see, if the insulin is necessary then don't think it is some drug with the last choice of treatment. If it is required, you should not refuse. You have to take.” (P-15, Male)*

b. **Self-diagnosing from the symptoms of friends and family members**

Even though patients had poor knowledge about western medication, doctors (*n*=5) described how their patients would often suggest the use of antidiabetic medications to family or friends, assuming that symptoms experienced by them were diabetes related. These actions led to reported adverse events as highlighted in this narrative.

*“A female elderly patient who was on antidiabetic medication observed her granddaughter eating 4-7 jalebies [Indian Sweet]. The patient thought that she will develop diabetes and gave half of the tablet [Glibenclamide] which she was taking for type 2 diabetes. Granddaughter started developing hypoglycemia...” (P-15, Male)*

c. **Stigma of diabetes**

Apart from poor knowledge about diabetes, many doctors (*n* = 11) expressed concerns that their patients were fearful of being diagnosed or labelled with diabetes due to the associated stigma. They mentioned that patients were hesitant to take antidiabetic medications in public and may skip medications for fear of being excluded by friends and family members.

*“…a lot of patients confide their disease only to their very close one...They don’t want to tell other people because they might be looked down upon…so a lot of patients do miss their medication when they go out for some functions [social events].” (P-09, Male)*

Due to this stigma, some doctors (n = 4) said that patients preferred lifestyle modifications and traditional medications over use of western medications.

*“Patients feel like they don’t want to be diagnosed with diabetes…when a newly detected diabetes patient comes to us, we will start with medications. But patients say, I will take Ayurveda [traditional medications] and proper diet and walking for few months.” (P-1, Female)*

d. **Doctors’ lack of trust on medications available under government generic medications scheme**

As many as 15 doctors felt that their efforts to facilitate the use of western medications were also hindered due to the lack of trust in the efficacy of medications and availability of fixed-drug combinations available under government subsidy genetic medications schemes such as the Jan Aushadhi Scheme (JAS).

*“Now most of them [patients] are going for Jan Aushadhi medications. But we don't know the quality of Jan Aushadhi medications. Almost 50% of Jan Aushadhi medication patients will not have controlled blood sugar.”(P-16, Male)*

As a result of these factors, doctors prefer to prescribe patients with fixed drug combination medications, which are often perceived to be unavailable under JAS.

*“There [Jan Aushadhi] you won’t get combined drugs. If you write glimepiride plus metformin fixed-drug combination, they will dispense separately and patients might miss one tablet to take. That is the reason patients will come with complications and we have to start with Insulin. The moment we start insulin patient will never come to me.” (P-01, Female)*

e. **Accessibility and affordability: barriers to adoption of western medications**

Limited access to healthcare was cited by most doctors (*n* = 17) as the key reason for not continuing with the prescribed western medications. For example, one doctor mentioned lack of timely assistance to administer insulin.

*“One elderly patient having ophthalmic problem [blurred visions]... Attenders say patient stays alone. One nurse stays next to patient [neighbour]. But she cannot ask nurse to help every time to administer insulin, because it should be given twice or thrice a day and they have to take food immediately. So, if the nurse is not available then it is a problem. So, I give insulin only if facilities are there.”(P-01, Female)*

Other barriers included transportation and distance from the healthcare service point and reliance on other family members. In addition, the cost of taking multiple tablets was noted as a barrier to western medications.

*“… So many elderly patients particularly from rural [areas say that] my son did not get tablets so I could not take tablets. So many times it happens. We have told them to take one tablet two times a day but they take half tablet. Because they could not buy the tablet or the tablet was not available.”(P-17, Male)*

It was felt that the easier access to internet likely led patients with type 2 diabetes to self-experiment with their condition and western medications.

*“Most of the times the elite patients…from urban area try to look for side effects even before starting the [western] medication. If we prescribe medications today, they postpone it and wait till symptoms worsen and then start medications. During that time they would have done all this Google and other things.”(P-18, Male)*

### Doctors reported little faith in traditional medicines

- **Contrasting beliefs in traditional medications between doctors and patients**

While it was suggested that most of their patients had great faith in the use of traditional medication, doctors expressed contrasting opinions. Many doctors (*n* = 15) disagreed with patients’ beliefs that traditional medication can cure diabetes with no adverse effects.

*“Patients wanted to get rid of diabetes without any side effects. There is a myth, allopathic [western] medications have side effects, it doesn't cure. Once you start medications you will be addicted to that. Because of this myth, they wanted to take a shortcut.” (P-14, Female)*

In addition, doctors (n = 14) felt that friends and family members were often influencing by providing information regarding the use of traditional medications.

*“People don't believe the doctor. They believe their friends, neighbours and people they meet in bus stops or railway stations or airports or house maid. They say, my house maid said so we went there [to take traditional medications].” (P-20, Male)*

Furthermore, there were concerns over misleading marketing strategies that traditional medication manufacturing companies used to sell diabetes-related products that influenced the likelihood of patients practicing self-medication.

*“There are some business people who sells this herbal products for treating diabetes. They make [health] camps and give patients a cup of herbal drinks and tell them to drink for 20-30 days. Make people to get used to that. I don’t know…what it contains.” (P-8 Male)*

b. **Doctors have less knowledge in traditional medicine**

While doctors’ opinions about traditional medications contrasted with patients, many acknowledged that they had limited knowledge in this area. Doctors (*n* = 8) suggested patients stop using traditional medications as they perceived themselves not to be experts in traditional medicine and were concerned about drug-to-drug interactions between the two systems.

*“If anybody is on alternative [traditional] medicines, I will honestly tell them that I am not a qualified person to comment because I have not studied that. What I know is only allopathic [western] medicines. Alternative [traditional] medicines will have a drug to drug interaction [with western medications]...So, I will tell them to stop all these drugs.”(P-10, Male)*

### Limited strategies implemented by doctors to overcome barriers to self-medication

Considering the above-mentioned factors influencing self-medication practices, doctors described strategies they use to manage their patients’ self-medication practices.

- **Doctors prescribed medications based on patient’s socioeconomic status**

Seventeen doctors prescribed medications based on the socioeconomic status of their patients. They prescribed fixed-drug combinations to those they perceived to be from more affluent backgrounds versus government funded medications to people from lower socioeconomic status.

*“Fixed-drug combinations are costly. So, I have to look what the patient can afford and what is the best medication, the lesser cost medication which is going to benefit the patient. So, yes, I do ask patients about their social, economic status and decide medications.” (P-09, Male)*

b. **Advice to continue both system of medications until the blood sugar levels are normal**

Half the doctors (*n* = 10) said that they could not deny the patient's choice in medications (traditional or western) and often advised their patients to continue with both systems of medications as long as their blood sugars were within the normal limits.

*“I don't deny the right to use alternative [traditional] medicine. I tell patients use medications what I have prescribed and along with alternative [traditional] medication…Idea is to control the sugars, doesn't matter how they control it.” (P-14, Female)*

c. **﻿Doctors insisted patients stop consuming traditional medications**

A few doctors (*n* = 8) had differing opinions and strongly encouraged their patients to cease using traditional medications and only consume western medications to manage diabetes.

*“I tell them you just follow [my advice]. I advise them to buy blood glucose monitoring…take the record and show the improvement. If you don’t show improvement, you ought to take my group of medicines…mine… allopathic [western] group of medicines. This is my strict advice to them.” (P-04, Male)*

Another male doctor said.

*“First of all, stop mixing up these alternate [traditional] systems of medication, it does not work. We do not expect a disease of 2019 to be treated with a medication of 17*^*th*^* Century.” (P-02, Male)*

d. **Doctors tried to provide health education to assist with their disease monitoring and progression**

A few doctors (*n* = 3) made efforts to educate their patients on how they could better manage their disease.

*“I will tell them there are three pillars for diabetes. First is medical nutritional therapy, second lifestyle modification and third is drugs. So, the first two steps I will take around 10 to 15 minutes and last is drugs. I tell them there is a possibility that this particular drug reduces your blood sugars. So, stop when you feel dizzy or anything like that. That they will keep in mind. The moment they come to know that their blood sugars is 70 mg/dl. They reduce the dose of tablets and meet me next time.”(P-17, Male)*

## Discussion

This qualitative was conducted to explore the doctors’ perceptions and understanding of the self-medication practices of people living with type 2 diabetes. It provided insights of doctors’ perceptions on the management of self-medication practices among people living with type 2 diabetes in India and the strategies implemented to overcome the factors that influence self-medication practices. Doctors in this study mentioned that their patients had greater trust on traditional medications due to perceived fewer adverse effects. But this perception among their patients had presented them with a challenge as their personal medicine practices contrast with this belief. Doctors in India primarily adopt a western medicine practices that have been largely influenced by the historical, social and political factors under the context of the continued colonial legacy of domination and hegemony of western ideals [28–30].

During the colonial rule in eighteenth and nineteenth century, western medicine was considered as superior and encouraged by the British whilst the traditional systems of medicine such as Ayurveda and Unani, received little patronage [29]. Little has changed since colonial rule as India continues to be influenced by western ideologies including in the field of medicine. Data from this study suggests that doctors worked within the confines of a western model of healthcare, had greater confidence in the efficacy of western over traditional medicine and were not willing to integrating other forms of practices by expressing concerns about the potential negative interactions between western and traditional medications [31, 32]. Due to colonial influence and greater trust in western medications, most doctors in this study reluctant to embrace their patients’ beliefs regarding traditional medications as a cure or for the management of type 2 diabetes. Therefore, there is a need develop more culturally sensitive healthcare environment which considers patients’ beliefs.

Despite adoption of western medical practices among doctors in India, recent approaches in medical practices such as the adoption of patient-centered approaches, where decisions related to health are determined through a discussion between doctors and patients, are absent in current models of care in India [33]. Studies found that people living with diabetes may experience emotional stress due to the complexity involved in the management of diabetes such as consuming medications, diet restrictions and physical activities [34, 35]. It is well known that patient-centered approaches improve the overall satisfaction of care, physical and social well-being and clinical practice of doctors by building a relationship that minimizes demographic, social and economic differences [36]. However, as is evident from the results of this study, decisions related to healthcare, are entirely determined by doctors. It may be due to the perceptions doctors have that their patients are lacking in knowledge related to diabetes management and the paternalistic medical model in India which dictates treatment to their patients with little regard for individual patient beliefs. Patient-centred care should be included in the medical curriculum so that doctors have opportunities to develop a better understanding of patients’ knowledge around diabetes management and facilitate an open conversation around the overall management of diabetes. Patient-centred care has been shown to encourage effective communication between doctors and patients when sharing health information and can help to build a trusting relationship with patients and their family members [37]. Additionally, the potential of adopting an integrative approach using a combination of traditional and western medicine in managing type 2 diabetes should also be considered as a potential solution to manage self-medication practices among people with diabetes.

Apart from doctors having to grapple with an ethos which was divergent from their own, there was recognition that broader social determinants affected prescription practices of western medications. Affordability of and access to medications were key barriers to prescription of western medications and were often perceived by doctors as reasons as to why people self-medicate. The lack of access to healthcare facilities in rural areas also compound the issues associated with access to medications. When doctors felt their patients were unable to access or afford branded medications, they prescribed generic medications available through the subsidized government pharmaceutical scheme, JAS. However, it appeared that doctors were also skeptical about the efficacy of medications available through this scheme. This prompted some to change their patients’ medications to the more expensive fixed-drug combinations of branded medications, citing greater trust in the efficacy of branded medications as well as lowering the pill burden. This perception does not appeared to be evidence-based as there are no current conclusive studies that have shown strong evidence to prove greater efficacy of branded medications when compared with generic medications [38–40]. This suggests that the skepticism among doctors related to the efficacy of generic medications available under JAS might be due to limited information on efficacy data and the misperceptions that less cost equals may equate to inferior quality [41, 42]. Greater efforts from the government is needed to address such concerns about JAS among doctors through the use of awareness campaigns and activities to improve the faith in JAS among doctors in India [41, 42].

Also, increasing the number of primary health centers as well as implementing strategies that would assist with medication administration e.g. nursing staff assisting with insulin may address part of these challenges [43]. In a bid to address this, the National Health Policy of India initiated “Ayushman Bharat Program” in 2018 to strengthen the policies and infrastructure of primary health centers by increasing the number of services offered, improved access to medications as well as considerations of integrating traditional medicine [44]. However, the impact of this program is yet to be evaluated.

## Strengths and Limitations

Inclusion of doctors working in both hospital and community settings with varying years of clinical experience gave strength to this study, improving the generalizability of our findings. The rigorous methodology used in this study in accordance with the COREQ guidelines strengthened the study results. Also, inclusion of an insider (RKP) and outsider (MK) to this study enabled the building of trust and rapport between the interviewer and participant. It enhanced the trustworthiness of the data analysis process. The lack of perspectives from the doctors in rural areas means that we were unable to explore if perceptions differed from those living in urban areas, potentially affecting the generalisability of these data. The inclusion of participants only from one south Indian city and minimal representation from female doctors may also affect the generalisability of the findings. However, fewer female doctors practice western medicine in India [45].

## Conclusion

This study provided insights to doctors’ understanding of their patients’ self-medication practices in managing type 2 diabetes in India. Doctors perceived that poor knowledge about the disease and western medications, increased trust among people in traditional medications and lack of access to and affordability of healthcare services are reasons for their patients’ self-medication practices. Therefore, this study suggests that there is a need to increase in the knowledge of JAS medications effectiveness amongst doctors and thereby garnering greater trust in generic medications for better healthcare delivery. Whilst also recognizing traditional forms of medicine, future studies should develop more strategies on how best to integrate traditional and western medicine into patient-centered care delivery.

## Supplementary Information

Below is the link to the electronic supplementary material.

Supplementary file1 (DOCX 29 KB)

## Data Availability

The data that supports the findings of this study are available in the supplementary material of this manuscript.
