# Supplementary material for: Using 17th century medication for modern diabetes management: Doctors’ perceptions of self-medication practices – A qualitative study
Source: J Diabetes Metab Disord. 2022 Nov 12;22(1):375–83. doi: 10.1007/s40200-022-01154-5 (PMC10225456; doi:10.1007/s40200-022-01154-5)
Supplement: Supplementary file 1 — Supplementary file1 (DOCX 29 KB) [file 40200_2022_1154_MOESM1_ESM.docx]

**Title:** Using 17th Century medication for modern diabetes management: Doctors’ perceptions of self-medication practices – A Qualitative Study.

**Name of the Journal:** Journal of Diabetes and Metabolic Disorders

**Author names and affiliations:**

Rahul Krishna Puvvada^a,b^, Clarice Y Tang^c^, Jency Thomas^a^, Mitch Kay^d^, Peter Higgs^d^, Markandeya Jois^a^, Madhan Ramesh^b^, Sabrina Gupta^d^

1. Department of Microbiology Anatomy Physiology and Pharmacology (MAPP), School of Agriculture Biomedicine and Environment (SABE), La Trobe University, Australia.
2. Department of Pharmacy Practice, JSS College of Pharmacy, JSS Academy of Higher Education and Research, Mysuru, Karnataka, India.
3. Department of Physiotherapy, School of Health Sciences, Western Sydney University, Australia.
4. Department of Public Health, School of Psychology and Public Health, La Trobe University, Australia.

**Address for Correspondence;**

Dr Sabrina Gupta, Lecturer, PhD,

Department of Public Health

School of Psychology and Public Health

La Trobe University, Melbourne

Email: S.Gupta@latrobe.edu.au

**Appendix 1: Semi-structured interview guide**

Doctor demographic details:

| **Study Title:** Social and cultural factors that influence self-medication habits of people living with type 2 diabetes in Mysuru, India | | |
| --- | --- | --- |
| Age: | | Gender: |
| Qualification: | | Designation: |
| Years / Months of experience: | | |
| Years / Months of experience in treating Type 2 Diabetes patients: | | |
| Do you attend regular training/conferences/ workshops? Yes or No:  If yes, how often: | | |
| How many patients do you see per day? |  | |
| How many Type 2 Diabetes patients do you see per day? |  | |
| How many newly diagnosed Type 2 Diabetes you see per day? |  | |
| How many self-medication practice patients in Type 2 Diabetes you see per day? |  | |

**Appendix 2: Semi-structured interview guide**

Can you tell me how long you have been working with people living with diabetes?

How many people do you see a week / month living with diabetes?

What proportion are new patients? (someone you have never seen before)?

Can you tell us what a standard consultation with a patient living with type 2 diabetes involves?

- What questions do you typically ask? (FHx, management, diagnosis)
- How many of these are regular follow up patients?
- How do you monitor the diabetes (e.g. HbA1c levels, FGT)?
- How many of the female patients have had GDM?
- Do you change your advice based on the patient’s background (e.g. socioeconomic status, literacy level)?
- What is your payment system like?
- How do patients find out about you?
- How do you manage their medications?
- How compliant/adherent do you think people are with their meds? Try to get a sense of the proportion of patients who are always, mostly, half half, rarely? Or tell us about the people you most worry about – get some detail here on what they are worried about etc
- Do you revise the medications they are on?
- How do manage self-medication behaviours?
- Where do you think they get their beliefs/ knowledge on these self-medicating practices?
- If patients are taking complementary or alternate medications (e.g. Ayurveda, natural remedies, homeopathy etc.), how do you get this information?
- How do you then manage this?

**Experiences with patients who self-medicate**

- Can you tell us about the barriers you face?
- Among these barriers, can you rank them in order of magnitude of the barrier. Explain why

**Consult alone or with the family**

- Describe how you would go about consulting with client’s with history of self-medication backgrounds
- What are your positive experiences when using family members as a part of the consultation process – any negative ones?

**Strategies for implementation**

- What strategies do you use to achieve behaviour changes in patients with self-medication practice?
- How do you assess health literacy levels?
- Do you assess them before presenting information? Test during sessions? What to see if patients are adherent/compliant?

**Appendix 3: Themes and Subthemes identified from the interviews**

| **Nodes** | **Subthemes** | **Themes** |
| --- | --- | --- |
| They may skip. Because that might have taken extra food so they take an extra dose or if they take food they may skip the tablet. That variability is there in some patients. (P-16, Male)  This happens in all chronic diseases not only in diabetes. If they develop some symptoms, the symptoms may be related to the disease or may not be related to the disease. But what they do is they relate it to the drug. Either they stop it or reduce the medication. (P-20, Male)  I saw 15 days back in the OPD. With 1 year old prescription that too patient came so confidently said mam last time I showed to you. I was like ok many times we will forget faces. Then I saw the OPD card and since one year no check up and nothing. Since one year no check-up for sugars just taking my old one year back prescription. (P-01, Female) | Types of self-medication practices | Doctors’ beliefs towards their patients’ use of traditional medicine and environmental factors influencing prescription practices |
| I usually try to find about what they have understood of the disease. Often people do not know the difference between type 1 and type 2 diabetes. Majority of them do not know about the disease. (P-02, Male)  I think 80% of newly diagnosed type 2 diabetes they hardly have any knowledge regarding complications of diabetes. (P-04, Male)  People are aware of diabetes, but they are not aware of why we should control of diabetes what complications can occur because of diabetes. They are not aware of that. (P-06, Male)  Most of the times patients don't understand diabetes. Fasting blood sugar check and they increase the dose. They don't understand that fasting is a reflection of previous day food. (P-18, Male) | Patients’ poor knowledge about the disease and medications |  |
| They say my uncle was consuming this tablet, so I came to know even I am diabetic and I have consumed this tablet. To know whether it is control or not under control. (P-17, Male)  A female elderly patient who was on antidiabetic medication observed her granddaughter eating 4-7 jalebies [Indian Sweet]. The patient thought that she will develop diabetes and gave half of the tablet [Glibenclamide] which she was taking for type 2 diabetes. Granddaughter started developing hypoglycemia...” (P-15, Male)  Friends or family members will tell patients that take this medication. This will be good for you. They will tell by just seeing the patients symptoms (P-11, Male) | Self-diagnosing from the symptoms of friends and family members |  |
| …a lot of patients confide their disease only to their very close one...They don’t want to tell other people because they might be looked down upon…so a lot of patients do miss their medication when they go out for some functions [social events]. (P-09, Male)  Patients feel like they don’t want to be diagnosed with diabetes…when a newly detected diabetes patient comes to us, we will start with medications. But patients say, I will take Ayurveda [traditional medications] and proper diet and walking for few months. (P-1, Female)  I have experienced diabetes people telling today I attended one function where I could not take the medicines in front of others, or I have not carried the medicines, or I cannot carried the insulin. (P-10, Male)  I think they feel very guilty that I am a diabetic that I am not supposed to eat so many things or this, if I take pill in front somebody oh! they will talk all these sick and complications or whatever that. They have the all that stupid imaginations. (P-04, Male) | Stigma of diabetes |  |
| Patient whom I know since many years it (medications from Jan Aushadhi scheme) didn’t work. I tell patients you give a try if it works for you then you continue with Jan Aushadhi medications. After one month [followup] if sugars are not controlling then I will completely stop Jan Aushadhi. Then I tell them to take my medications only. (P-01, Male)  Efficacy is not same it is not same there [medications available under Jan Aushadhi Scheme]. Especially, Insulin is not same. It is not same as efficacious as insulin available under brands. (P-03, Male)  I am not happy, not even with one single patient I am happy with Jan Aushadhi medications, very honest my opinion. (P-04, Male)  No, I don’t give, I don’t tell them to buy [JAS medications]…I will tell them strictly to go to the branded medication. Because I always prescribe fixed drug combinations, it is easy to take actually. Because most of the patient require multiple medications. So, if we add 5-6 or 10 drugs patient will get will not take properly. So, in fixed drug combinations we can give multiple medications in one tablet. (P-12, Male)  I cannot comment on that. Because the quality I can just see the sheet of medications which are sold. So, I cannot really comment on that. I don't have any evidence to comment on that. (P-18, Male) | Doctors’ lack of trust on medications available under government generic medications scheme |  |
| One elderly patient having ophthalmic problem [blurred visions]... Attenders say patient stays alone. One nurse stays next to patient [neighbour]. But she cannot ask nurse to help every time to administer insulin, because it should be given twice or thrice a day and they have to take food immediately. So, if the nurse is not available then it is a problem. So, I give insulin only if facilities are there. (P-01, Female)  So many elderly patients particularly from rural [areas say that] my son did not get tablets so I could not take tablets. So many times it happens. We have told them to take one tablet two times a day but they take half tablet. Because they could not buy the tablet or the tablet was not available. (P-17, Male)  I have seen some patients in medical camp. They say lack of medical facilities nearby. So, did not buy medications. (P-14, Female)  Yes, they [patients] have access to medications. Insulin in rural areas it is bit difficult. But somehow, they purchase in city. Again, convincing them for insulin is big challenge. They will say there is nobody to give insulin and it is not easily available. To start insulin among rural patients is big challenge. It takes lot of time in counselling these patients. (P-19, Male) | Accessibility and affordability: barriers to adoption of western medications |  |
| Because of cost or people will say it [western medications] has side effects and becomes costlier then they [patients] will stop this [western medications] and start taking ayurvedic [traditional] medications. (P-01, Female)  I prescribe only the allopathic [western] medications. But patients will ask some their friends who has taken allopathic [western medications] drugs and Ayurvedic medicine and traditional herbs [traditional medications]. Then they also mix it. (P-10, Male)  Family members or neighbours they say these [traditional medications] are good for you and will say that allopathic [western] medications have side effects. (P-11, Male)  People will always feel very attractive to alternative [traditional] medications because they are non-side effect. (P-12, Male)  People are scared of allopathic [western] medications because of side effects that's why they tried to you prefer other branch of medications [traditional medications] thinking they are free from side effects. (P-14, Female) | Contrasting beliefs in traditional medications between doctors and patients | Doctors reported little faith in traditional medicines |
| I can say I am better in allopathy because I know about the allopathy [western medicine] but I don’t do anything about Ayurveda [traditional medicine]. (P-11, Male)  Most of the time we do not even know because we cannot tell whether to continue or not [traditional medicine along with western medicine]. Because we are not expert in that herbal [traditional] medicine. (P-13, Male)  I will tell patients that I am not expert on Ayurvedic [traditional] medicine or homeopathic. So, it is up to them [patients] to take it [traditional medications] or not. (P-13, Male)  I tell them frankly that I don't know anything about alternative [traditional] medications. Because I don't know anything about the pharmacology or pharmacodynamics and how they interact with allopathic [western] medications. (P-19, Male) | Doctors have less knowledge in traditional medicine |  |
| I ask them [patients] can you afford going for this medicine which will try to bring down [blood sugars] very well. Those who can’t afford I use metformin and glipizide. Which are the cheapest medications available. (P-04, Male)  For poor social economic status people we recommend to go to Jan Aushadhi medications. (P-10, Male)  Before prescribing any medication, I just don’t prescribe medications like that…I will try to find weather patient can able to buy those medications and we will ask their profession, we go little bit deeper to know the socio economic status because it [diabetes] can cause economic burden, physical burden and mental burden. So, we have to go through all the parameters. (P-11, Male) | Doctors prescribed medications based on patient’s socioeconomic status | Limited strategies implemented by doctors to overcome barriers to self-medication |
| If they [patients] are taking self-medications like fenugreek seeds [traditional medications] which are commonly used. It is good for health, but I tell them [patients] to continue our medications [western medications]. As they have lot of benefits to health, but they are not totally enough to treat. They are helpful. I have seen many patients only on diet and taking self-medications but only it should be in the initial stages [of diabetes]. They do not work once patient has uncontrolled sugars. We will tell you have to take my [western] medications. (P-01, Female)  Yeah, some patients try and they have lot of belief on alternate [traditional] medicine. So, sometimes if they are taking [traditional] medication and if they want to continue that [traditional medications] then we will give our [western] medications and tell them continue both (P-05, Male)  Home remedies or Ayurveda [Traditional Medications] we won’t actually tell them they are not good. We are not against anything. We tell them to use what you feel better, but at the end blood sugars has to come down. (P-11, Male)  So, I tell them [patients] it is okay if you are taking [traditional medications]…no problem or any medicine some doctor only has given even though it is not allopathy. I will ask them [patients] to take but the sugars are not in control then you have to stop it. (P-13, Male) | Advice to continue both system of medications until the blood sugar levels are normal |  |
| I have seen many patients only on diet and taking self-medications [traditional medications]…but it is not going to help you in managing the diabetes. I tell them [patients] you have to take my [western] medications. (P-01, Female)  I support allopathic [western] medications. So I tell them please follow this advice, I am not sure whether you are on genuine Ayurvedic [traditional] medication (P-09, Male)  We will tell them [patients] to stop…Don’t take these medicines [traditional medications]. We cannot take both. Because I am not expert on Ayurvedic medicine or homeopathic. (P-13, Male) | Doctors insisted patients stop consuming traditional medications |  |
| They [Patients] will tell allopathic [western] medicines will have side effects. I tell them even a small simple tablet for cold or fever has its own side effect. Anti-diabetic medications are also having some side effects. All these side effects we will explain to the patient and we will tell them about the hypoglycaemia. We tell them to see any side effects and report to us if they notice. (P-10, Male)  I tell them the side effects of allopathic medications even before the start taking medications. These are the ones you can expect. So, if any of these side effects appear you please come back. I tell them if there is any problem there should be a solution...You don't need to stop the medications. For example, metformin can cause gastritis. In those cases, we will reduce the dose of metformin can increase its dose gradually or we can add proton pump inhibitors. So, there is always a solution for it. (P-14, Female) | Doctors tried to provide health education to assist with their disease monitoring and progression |  |
